# An Evidence-Based Multidisciplinary Approach Focused on Creating Algorithms for Targeted Therapy of Infection-Related Ventilator-Associated Complications (IVACs) Caused by *Pseudomonas aeruginosa* and *Acinetobacter baumannii* in Critically Ill Adult Patients

**DOI:** 10.3390/antibiotics11010033

**Published:** 2021-12-28

**Authors:** Milo Gatti, Bruno Viaggi, Gian Maria Rossolini, Federico Pea, Pierluigi Viale

**Affiliations:** 1 Department of Medical and Surgical Sciences, Alma Mater Studiorum University of Bologna, 40138 Bologna, Italy; milo.gatti2@unibo.it (M.G.); pierluigi.viale@unibo.it (P.V.); 2 SSD Clinical Pharmacology, Department for Integrated Infectious Risk Management, IRCCS Azienda Ospedaliero-Universitaria di Bologna, 40138 Bologna, Italy; 3 Neurointensive Care Unit, Department of Anesthesiology, Careggi University Hospital, 50134 Florence, Italy; bruno.viaggi@gmail.com; 4 Department of Experimental and Clinical Medicine, University of Florence, 50134 Florence, Italy; gianmaria.rossolini@unifi.it; 5 Microbiology and Virology Unit, Florence Careggi University Hospital, 50134 Florence, Italy; 6 IRCCS Fondazione Don Carlo Gnocchi, 50143 Florence, Italy; 7 Infectious Diseases Unit, Department for Integrated Infectious Risk Management, IRCCS Azienda Ospedaliero-Universitaria di Bologna, 40126 Bologna, Italy

**Keywords:** antimicrobial stewardship, infection-related ventilator-associated complications, non-fermenting Gram-negative pathogens, multidisciplinary taskforce, PK/PD dosing optimization, targeted antibiotic therapy

## Abstract

(1) Background: To develop evidence-based algorithms for targeted antibiotic therapy of infection-related ventilator-associated complications (IVACs) caused by non-fermenting Gram-negative pathogens. (2) Methods: A multidisciplinary team of four experts had several rounds of assessments for developing algorithms devoted to targeted antimicrobial therapy of IVACs caused by two non-fermenting Gram-negative pathogens. A literature search was performed on PubMed-MEDLINE (until September 2021) to provide evidence for supporting therapeutic choices. Quality and strength of evidence was established according to a hierarchical scale of the study design. Six different algorithms with associated recommendations in terms of therapeutic choice and dosing optimization were suggested according to the susceptibility pattern of two non-fermenting Gram-negative pathogens: multi-susceptible *Pseudomonas aeruginosa* (PA), multidrug-resistant (MDR) metallo-beta-lactamase (MBL)-negative-PA, MBL-positive-PA, carbapenem-susceptible *Acinetobacter baumannii* (AB), and carbapenem-resistant AB. (3) Results: Piperacillin–tazobactam or fourth-generation cephalosporins represent the first therapeutic choice in IVACs caused by multi-susceptible PA. A carbapenem-sparing approach favouring the administration of novel beta-lactam/beta-lactamase inhibitors should be pursued in the management of MDR-MBL-negative PA infections. Cefiderocol should be used as first-line therapy for the management of IVACs caused by MBL-producing-PA or carbapenem-resistant AB. Fosfomycin-based combination therapy, as well as inhaled colistin, could be considered as a reasonable alternative for the management of IVACs due to MDR-PA and carbapenem-resistant AB. (4) Conclusions: The implementation of algorithms focused on prompt revision of antibiotic regimens guided by results of conventional and rapid diagnostic methodologies, appropriate place in therapy of novel beta-lactams, implementation of strategies for sparing the broadest-spectrum antibiotics, and pharmacokinetic/pharmacodynamic optimization of antibiotic dosing regimens is strongly suggested.

## 1. Introduction

Infection-related ventilator-associated complications (IVACs) represent the most prevalent infective events in patients admitted to the intensive care unit (ICU) and requiring mechanical ventilation [1], accounting approximately for one-third of hospital-acquired pneumonia (HAP) cases [2]. IVACs are associated with high mortality rate (over 50%) and with a remarkable impact on length of ICU stay, antibiotic use, and overall health care costs [2,3,4,5]. Gram-negative pathogens are responsible for the majority of HAP and ventilator-associated pneumonia (VAP), and among these, the non-fermenting Gram-negative pathogens (especially *Pseudomonas aeruginosa* and *Acinetobacter baumannii*) are responsible for a remarkable amount of IVACs in critically ill patients, second only to Staphylococcus aureus in terms of prevalence [6,7].

*Pseudomonas aeruginosa* and *Acinetobacter baumannii* are both characterized by innate resistance mechanisms against multiple antimicrobials. Furthermore, these pathogens may easily acquire new resistances by different mobile elements, thus making extremely challenging the choice of appropriate antibiotic therapy in this setting [6,7,8,9]. Prompt initiation of empirical broad-spectrum antibiotic treatment is necessary in critically ill patients with suspected IVAC, including agents with activity against non-fermenting Gram-negative pathogens according to the existence of specific risk factors or in case of epidemiological settings characterized by high prevalence. In the last report on antimicrobial surveillance issued by the European Center for Disease Prevention and Control (ECDC) [10], the proportion of invasive isolates of multidrug resistant (MDR) *Pseudomonas aeruginosa* and carbapenem-resistant *Acinetobacter baumannii* accounted for 12.1% and 32.6%, respectively. In the Italian context, the proportion of the invasive strains of MDR *Pseudomonas aeruginosa* is in line with European data (13.1%), while that of carbapenem-resistant *Acinetobacter baumannii* isolates accounts for as much as 80% [10]. However, once that the causative pathogen has been identified and its susceptibility pattern has been defined, therapy should be revised and targeted, as recommended by the Surviving Sepsis Campaign guidelines [11].

Microbiological confirmation of IVAC represents a crucial step for enabling targeted antibiotic therapy in critically ill patients. Unfortunately, traditional culture-based methods are time-consuming as they often require at least 1–2 days for pathogen isolation and an additional day for determination of the antibiotic susceptibility pattern. Furthermore, culture-based methods exhibit low sensitivity, as they may provide pathogen identification in less than 40% of patients with clinically diagnosed IVACs [12]. This has traditionally made challenging and often difficult a rapid implementation of targeted antibiotic therapy [10]. In recent years, the development of rapid molecular tests has revolutionized microbiological diagnosis in the pneumonia setting. These tests, based on syndromic panels, may provide fast identification of the respiratory pathogens coupled with detection of relevant genotypic markers of resistance. This approach is expected to have a huge impact in daily clinical practice, by making more and more applicable both targeted antibiotic therapy and antimicrobial stewardship strategies [12,13].

Appropriate targeted antimicrobial therapy, coupled with antibiotic dose optimization and implementation of antimicrobial stewardship programs, could simultaneously maximize efficacy, reduce antibiotic overconsumption, and minimize the development of resistance in ICU patients [14]. This could be achieved by means of the coordinated approach of a multidisciplinary task force, composed by the intensive care physician, the infectious disease consultant, the clinical microbiologist, and the MD or PharmD clinical pharmacologist. Early diagnosis with prompt identification of the causative pathogen of IVACs coupled with targeted antibiotic therapy based on antimicrobial susceptibility testing and on dosing optimization according to the pharmacokinetic/pharmacodynamic (PK/PD) concepts and the therapeutic drug monitoring (TDM)-guided approach may be fundamental for this purpose.

This multidisciplinary opinion article aims to develop evidence-based algorithms for targeted antibiotic therapy of IVACs caused by two non-fermenting Gram-negative pathogens in critically ill adult patients. The objective is to provide a useful guidance for intensive care physicians either in appropriately placing novel antimicrobial agents in lack of definitive evidence or in considering antimicrobial stewardship strategies for possibly sparing the broadest-spectrum antibiotics.

## 2. Results

### 2.1. Targeted Treatment of IVACs Caused by Pseudomonas aeruginosa in Critically Ill Adult Patients

**Figure 1:**
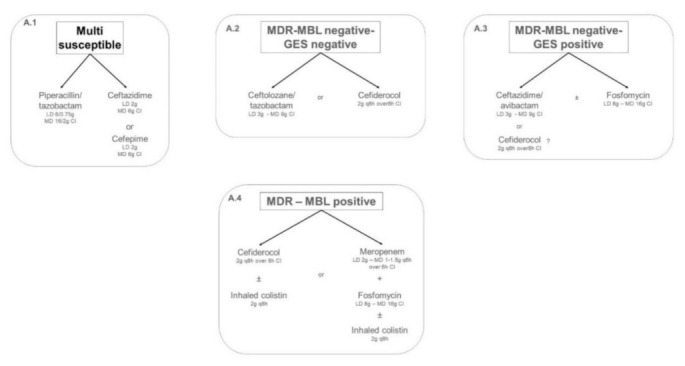
Algorithms for targeted treatment of IVAC caused by *Pseudomonas aeruginosa* with different pattern of antibiotic susceptibility. CI: continuous infusion; EI: extended infusion; LD: loading dose; MBL: metallo-beta-lactamase; MD: maintenance dose; MDR: multidrug resistance.

Therapeutic algorithm for targeted treatment of IVACs caused by Pseudomonas aeruginosa in adult ICU patients is shown in Figure 1.

#### 2.1.1. Multi-Susceptible *Pseudomonas aeruginosa*

Continuous infusion piperacillin–tazobactam (18 g/day after 6.75–9 g loading dose [LD]) is recommended as target therapy for the management of IVACs caused by multi-susceptible *Pseudomonas aeruginosa*. Ceftazidime (6–8 g/day after 2 g LD) or cefepime (2 g LD followed by 6 g/day) in continuous infusion should be reserved as second-line alternatives in case of clinical or microbiological failure (Figure 1, panel A.1). ATS/IDSA guidelines [15] recommended the use of piperacillin–tazobactam, ceftazidime or cefepime as definitive therapy of HAP or VAP requiring a coverage on *Pseudomonas aeruginosa* with demonstrated susceptibility, suggesting the administration in EI or CI to maximize lung exposure. A summary of the studies evaluating the efficacy of piperacillin–tazobactam or third/fourth-generation cephalosporins in patients affected by IVACs caused by multi-susceptible *Pseudomonas aeruginosa* is provided in Table 1.

Two different RCTs investigated the efficacy of piperacillin–tazobactam in the management of IVACs caused by *Pseudomonas aeruginosa* [16,17]. Jaccard et al. [16] randomized 371 patients affected by nosocomial infections (49.2% HAP, of which 28% caused by *Pseudomonas aeruginosa*) to piperacillin–tazobactam or imipenem–cilastatin. Although no difference in clinical failure rate (17.0% vs. 29.0%; *p* = 0.09) and mortality rate (8% vs. 9%; *p* = 0.78) was found between the two agents in HAP subgroup, a significantly lower clinical failure rate was found in patients affected by HAP due to *Pseudomonas aeruginosa* receiving piperacillin–tazobactam compared to imipenem–cilastatin (10.0% vs. 50.0%; *p* = 0.004). Notably, a significantly higher rate of resistance development was reported with imipenem–cilastatin compared to piperacillin–tazobactam in HAP due to *Pseudomonas aeruginosa*. Joshi et al. [17] randomized 300 patients (87% HAP, of which 7.7% due to *Pseudomonas aeruginosa*), reporting a significantly higher clinical cure rate in 155 patients receiving piperacillin–tazobactam associated with tobramycin compared to 145 patients treated with ceftazidime plus tobramycin (74.2% vs. 57.9%; *p* = 0.004). Furthermore, a trend to higher microbiological eradication was found in *Pseudomonas aeruginosa* subgroup with piperacillin–tazobactam (67% vs. 30%; *p* = 0.19). Babich et al. [18] retrospectively evaluated clinical outcome in 767 patients receiving definitive monotherapy for the treatment of BSI due to *Pseudomonas aeruginosa*. No difference in 30-day mortality rate emerged between ceftazidime (OR 1.14; 95% CI 0.52–2.46) or piperacillin–tazobactam (OR 1.30; 95% CI 0.67–2.51) compared to carbapenems (meropenem or imipenem) at propensity score analysis, while a higher development of new resistance in *Pseudomonas aeruginosa* isolates occurred in patients treated with carbapenems compared to ceftazidime or piperacillin–tazobactam (17.5% vs. 12.4% vs. 8.4%; *p* = 0.007). However, no subgroup analysis according to IVACs was performed.

Several observational studies recently investigated the role of ceftazidime and cefepime in the management of *Pseudomonas aeruginosa* BSIs [18,19,20]. Rate of IVACs ranged from 14.7% to 30% and ICU admission was reported in up to 32.2% of cases. Conflicting findings emerged in retrospective studies performing a subgroup analysis according to MIC of *Pseudomonas aeruginosa* for ceftazidime or cefepime [19,20]. Su et al. [19] found that a MIC for cefepime ≥ 4 mg/L may predict an unfavourable outcome among patients with serious infections due to *Pseudomonas aeruginosa*, including 30% of HAP/VAP. Conversely, Ratliff et al. [20] found no difference in mortality rate between patients exhibiting a MIC for ceftazidime or cefepime ≤ 2 mg/L compared to those with a MIC > 2 mg/L in *Pseudomonas aeruginosa* BSIs (17.2% vs. 27.6%; *p* = 0.34), although subgroup analysis in subjects affected by IVACs was not performed.

In regard to recommended dosages, some well-established evidence may support the use of CI over intermittent infusion in administering traditional antipseudomonal beta-lactams in critically ill patients [21]. Additionally, we recently showed in a large cohort of critically ill patients having documented Gram-negative infections treated with CI traditional beta-lactams that early achievement of an aggressive PK/PD target of Css/MIC > 5 within the first 72 h was significantly associated with both microbiological eradication and prevention of resistance development [22]. In the same study, *Pseudomonas aeruginosa*-related infections were independently associated with higher risk of microbiological failure [22]. Accordingly, we consider that when treating IVACs caused by multi-susceptible *Pseudomonas aeruginosa* isolates, the use of CI piperacillin–tazobactam and/or ceftazidime, and/or cefepime after loading may represent a valuable approach for rapidly achieving and maintaining an aggressive PK/PD target helpful at achieving microbiological eradication.

#### 2.1.2. Multidrug-Resistant (MDR) Metallo-Beta-Lactamase (MBL)-Negative *Pseudomonas aeruginosa*

The emergence of MDR and extensively drug-resistant (XDR) *Pseudomonas aeruginosa* is a major clinical concern. The underlying mechanisms of the MDR/XDR phenotype are heterogeneous and can be mediated by the selection of mutations in the chromosomal genes or by horizontal acquisition of resistance determinants, including beta-lactamase and carbapenemase genes [23]. A major distinction regards MDR/XDR *Pseudomonas aeruginosa* isolates producing or not metallo-beta-lactamases. In case of MDR/XDR metallo-beta-lactamases (MBL)-negative isolates, antibiotics should be chosen according to antimicrobial susceptibility tests. Among the different beta-lactamases responsible for MDR/XDR phenotype, GES enzymes, belonging to class A carbapenemases, could play a remarkable role in the selection of appropriate targeted anti-pseudomonal agents. GES enzymes represents a major resistance mechanism of MDR/XDR high-risk clones (e.g., ST-235 highly virulent clone), exhibiting higher virulence compared to other clones (e.g., ST111 or ST175) and resulting in more severe acute infections with significant mortality [23]. Furthermore, production of GES enzymes confers resistance to ceftolozane–tazobactam [23]. In case of IVACs caused by MDR/XDR MBL-negative GES-negative isolates (Figure 1, Panel A.2), prolonged infusion of ceftolozane–tazobactam (3 g q8h CI after 3 g LD) is recommended as first-line therapy. Cefiderocol (2 g LD followed by 2 g q8h in CI) should be reserved as second-line alternative in case of clinical or microbiological failure (Figure 1, Panels A.2-A.3). A summary of the studies evaluating the efficacy of ceftolozane–tazobactam or cefiderocol in patients affected by IVACs caused by MDR GES-negative *Pseudomonas aeruginosa* is provided in Table 2.

In a phase III RCT, Kollef et al. [24] reported no significant difference in mortality (25.4% vs. 18.5%; *p* = NS) and clinical cure rate (57.1% vs. 60%) between ceftolozane–tazobactam and meropenem in subgroup of critically ill patients affected by HAP or VAP caused by MDR/XDR *Pseudomonas aeruginosa*. However, *Pseudomonas aeruginosa* accounted only for 17.4% of overall isolates. Several observational studies and case series [25,26,27,28,29,30,31,32,33,34,35,36,37,38,39,40] reported the high efficacy of ceftolozane–tazobactam in critically ill patients (ICU admission ranging from 23.8% to 100%) affected by HAP/VAP in most cases, although both relapse and resistance rate were not negligible (respectively up 29% and 14%). Notably, Pogue et al. [25] retrospectively analysed two hundred MDR *Pseudomonas aeruginosa* (64% HAP/VAP) infections, 100 treated with ceftolozane–tazobactam and the other 100 managed with polymyxin- or aminoglycoside-based regimen. A significant higher clinical cure rate (81% vs. 61%; *p* = 0.002) and lower AKI occurrence (6% vs. 34%; *p* < 0.001) was found in patients treated with ceftolozane–tazobactam. Similarly, Fernandez-Cruz et al. [28] found a significant lower mortality rate in 19 patients treated with ceftolozane–tazobactam compared to 38 subjects receiving other anti-pseudomonal agents (5.4% vs. 28.9%; *p* = 0.045) for infections caused by ST-175 *Pseudomonas aeruginosa* clones. All patients were affected by haematological malignancies and rate of nosocomial pneumonia was 26.3%. Interestingly, Gallagher et al. [29] retrospectively analysed 205 patients affected by severe *Pseudomonas aeruginosa* infection (51.2% ICU admission; 59% HAP/VAP) and treated with ceftolozane–tazobactam, reporting that pneumonia was significantly associated with lower microbiological cure than observed for other infection sites (OR 0.12; 95% CI 0.05–0.30). Rodriguez-Nunez et al. [30] assessed 90 critically ill patients affected by lower respiratory tract infections due to MDR/XDR *Pseudomonas aeruginosa* treated with ceftolozane–tazobactam, founding that a MIC > 2 mg/L was an independent predictor of mortality at multivariate analysis.

Two different phase III RCTs [41,42] supported the efficacy of cefiderocol for the treatment of IVACs caused MDR *Pseudomonas aeruginosa*, reporting no significant difference in mortality or clinical cure rate compared to meropenem or best available therapy (including combination of aminoglycoside, carbapenems, colistin, fosfomycin or tigecycline) in case of carbapenem-resistant isolates. However, the proportion of carbapenemase-producer isolates in these studies was only 8–15%. Several in vitro studies [43,44,45] demonstrated the activity of cefiderocol against MDR (carbapenemase-negative meropenem non-susceptible) *Pseudomonas aeruginosa* (MIC range 0.002–8; MIC50 0.12). Notably, high susceptibility (90%) against GES-positive isolates was reported.

In regard to recommended dosages, the use of CI for ceftolozane–tazobactam is supported by the findings of a prospective study showing that among 72 patients affected by MDR *Pseudomonas aeruginosa* infections (mainly VAP), CI was associated with a higher probability of target attainment of the aggressive PK/PD target of 100% *f*T > 4 × MIC [46]. Additionally, a case-control study evaluating 28 patients affected by MDR *Pseudomonas aeruginosa* infections (67.9% pneumonia) showed that prolonging infusion up to 3 h allowed significantly lower resistance development compared to intermittent infusion (0.0% vs. 58.3%; *p* = 0.04) [47]. Consequently, we suggest CI of ceftolozane–tazobactam after loading for rapidly achieving and then maintaining aggressive PK/PD targets in IVACs caused by MDR MBL-negative GES-negative *Pseudomonas aeruginosa* isolates.

Continuous infusion of ceftazidime–avibactam (7.5 g/day after 2.5 LD) or cefiderocol (2 g LD followed by 2 g q8h in CI) in monotherapy or in association with fosfomycin (6–8 g LD followed by 16 g/day CI) represents the first-line therapy for the management of IVACs caused by MDR GES-positive *Pseudomonas aeruginosa* (Figure 1, Panel A.3). A summary of the evidence assessing the efficacy of ceftazidime–avibactam alone or with fosfomycin in patients affected by IVACs caused by MDR GES-positive *Pseudomonas aeruginosa* is provided in Table 3.

In a phase III RCT [48], Torres et al. reported no significant difference in clinical cure rate (64.3% vs. 77.1%; *p* = NS) between ceftazidime–avibactam and meropenem in subgroup of critically ill patients affected by HAP or VAP caused by MDR *Pseudomonas aeruginosa*. However, *Pseudomonas aeruginosa* accounted only for 30% of overall isolates, and no further analysis was provided to identify resistance mechanisms of *Pseudomonas aeruginosa* isolates (including carbapenemases/GES-production). Several observational studies and case series/reports [49,50,51,52,53] demonstrated the efficacy of ceftazidime–avibactam in critically ill patients (ICU admission ranging from 41.5% to 100%) affected by HAP/VAP in most cases. Particularly, Vena et al. [50] reported a clinical success of 87.8% in *Pseudomonas aeruginosa* infections (48.8% HAP/VAP) in 41 patients treated with ceftazidime–avibactam (prolonged infusion in 36.6% of cases). Notably, an in vitro analysis of a retrospective study including 24 patients affected by XDR *Pseudomonas aeruginosa* infections (33.3% nosocomial pneumonia) found an overall susceptibility to ceftazidime–avibactam of 100% in GES-5-positive strains belonging to ST235 clone, showing a MIC_90_ of 6 mg/L [54].

Only preclinical evidence supported the association therapy between ceftazidime–avibactam and fosfomycin against MDR *Pseudomonas aeruginosa* [55,56,57]. Synergism between the two agents was retrieved in 25–61.9% of isolates and was also confirmed in a murine model of infection [55].

Cefiderocol could represent a valuable alternative to ceftazidime–avibactam in the treatment of IVACs caused by MBL-negative MDR GES-positive *Pseudomonas aeruginosa*, but caution is required due to the limited clinical experience in this setting and the quite low number of GES-positive strains that have been tested in vitro for susceptibility to this antibiotic. Anyway, considering that in most clinical studies MBL-negative MDR GES-positive *Pseudomonas aeruginosa* isolates were rarely characterized, caution is recommended in choosing any agent, including ceftazidime–avibactam.

In regard to recommended dosages, evidence supporting the use of CI for ceftazidime–avibactam is stemmed from a large observational study among 577 patients with KPC-producing *Klebsiella pneumoniae* treated with ceftazidime–avibactam, in which prolonged and/or CI was independently associated with higher survival rate compared to intermittent infusion [58]. In regard to cefiderocol, we recently showed in a descriptive case series of PK/PD target attainment and microbiological outcome in critically ill patients with documented severe XDR *Acinetobacter baumannii* BSI and/or VAP treated with cefiderocol that the standard 3 h infusion was ineffective in achieving the aggressive PK/PD of 100%fT > 4 × MIC in more than 50% of included patients. This resulted in a remarkable high rate of microbiological failure, especially among VAP cases [59].

Consequently, we recommend CI of ceftazidime–avibactam and/or of cefiderocol after loading for rapidly achieving and then maintaining aggressive PK/PD targets in IVACs caused by MDR *Pseudomonas aeruginosa*, also taking into account the limited ELF penetration rate of these agents [60,61,62].

#### 2.1.3. Multidrug-Resistant (MDR) Metallo-Beta-Lactamase (MBL)-Positive *Pseudomonas aeruginosa*

Prolonged infusion of cefiderocol (2 g LD followed by 2 g q8h in EI or CI) in combination therapy with inhaled colistin (2 MU q8h) is recommended as first-line therapy for the management of IVACs caused by MDR metallo-beta-lactamase-producers (VIM, IMP, or NDM) *Pseudomonas aeruginosa*. Combination therapy including high-dose prolonged infusion meropenem (1–1.5 g q6h after 2 g LD), fosfomycin (6–8 g LD followed by 16 g/day CI), and inhaled colistin (2 MU q8h) should be reserved as second-line alternative (Figure 1, panel A.4). A summary of the evidence assessing the efficacy of these antibiotic regimens in patients affected by IVACs caused by MDR metallo-beta-lactamase-positive *Pseudomonas aeruginosa* is provided in Table 4.

Currently, no case of IVACs caused by metallo-beta-lactamase-positive *Pseudomonas aeruginosa* treated with cefiderocol exists. Real-world evidence is limited to two different case reports showing the efficacy of cefiderocol in osteomyelitis caused by NDM- or VIM-positive *Pseudomonas aeruginosa* [66,67]. In vitro studies [44,45,63] demonstrated the activity of cefiderocol against MBL-producing *Pseudomonas aeruginosa*, showing an overall susceptibility respectively of 93.3%, 80%, and 45.5% for VIM-, IMP-, or NDM-positive strains [44]. For VIM-positive isolates, MIC ranged from 0.008–0.03 to 1–2 mg/L with a MIC_50_ of 0.25 [45,63].

Although no clinical evidence for the use of meropenem in combination with fosfomycin for the management of IVACs caused by MBL-producing *Pseudomonas aeruginosa* currently exists, Albiero et al. [64] reported in vitro the synergic effect of the combination regimen in ten MBL-positive *Pseudomonas aeruginosa*. Synergism was found in 100% of isolates, resulting in a median decrease of MIC50 and MIC90 by eight-fold. PK/PD simulation showed that high-dose fosfomycin (6–8 g q8h) or meropenem (1.5 g q6h in 3 h EI) achieved the probability of target attainment of ≥ 90% respectively at an MIC of 32 mg/L and 16 mg/L. Additionally, combination therapy resulted in a significantly increase in the cumulative fraction rate against MBL-positive *Pseudomonas aeruginosa* compared to monotherapy with meropenem (32% vs. 68%) or fosfomycin (0% vs. 74%) [64].

A systematic review including patients affected by HAP or VAP due to MDR *Pseudomonas aeruginosa* reported respectively a clinical success and microbiological eradication in 70.4% and 71.3% of cases with inhaled colistin monotherapy [65]. No evidence for inhaled colistin in association with cefiderocol or meropenem/fosfomycin combination therapy currently exists.

In regard to recommended dosages, evidence supporting the use of high-doses CI meropenem after 2 g loading stemmed from a recent study that assessed the impact of maximizing Css/MIC ratio on efficacy of CI meropenem against documented Gram-negative infections in critically ill patients [68]. In that study, it was shown that a Css/MIC ratio ≥4.63 was significantly associated with favourable clinical outcome among 74 ICU patients [68]. Monte Carlo simulation showed that higher meropenem dosages by CI should be recommended for dealing tackling appropriately *Pseudomonas aeruginosa* and *Acinetobacter baumannii* infections in critically ill patients with preserved renal function [68]. Likewise, a similar aggressive PK/PD target of Css/MIC > 5 within the first 72 h was significantly associated with both microbiological eradication and prevention of resistance development in a large cohort of critically ill patients having documented Gram-negative infections treated with CI traditional beta-lactams [22]. In regard to cefiderocol, we recently showed in a descriptive case series of PK/PD target attainment and microbiological outcome in critically ill patients with documented severe XDR *Acinetobacter baumannii* BSI and/or VAP that treatment with cefiderocol at the standard 3 #h infusion was ineffective in achieving the aggressive PK/PD of 100%fT > 4 × MIC in more than 50% of included patients. This resulted in a remarkable high rate of microbiological failure, especially among VAP cases [59]. Consequently, we suggest CI of cefiderocol or of high dose meropenem after loading for promptly achieving and then maintaining an aggressive PK/PD target in critically ill patients affected by IVACs due to MDR *Pseudomonas aeruginosa*.

### 2.2. Targeted Treatment of IVACs Caused by Acinetobacter baumannii in Critically Ill Adult Patients

**Figure 2:**
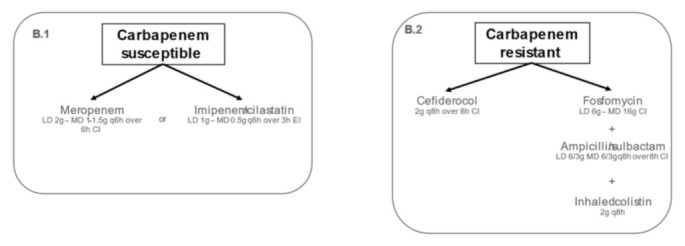
Algorithms for targeted treatment of IVAC caused by full-susceptible and multidrug-resistant *Acinetobacter baumannii*. CI: continuous infusion; EI: extended infusion; LD: loading dose; MD: maintenance dose; MDR: multidrug resistance.

Therapeutic algorithm for targeted treatment of IVACs caused by *Acinetobacter baumannii* in adult ICU patients is shown in Figure 2.

#### 2.2.1. Carbapenem-Susceptible *Acinetobacter baumannii*

Continuous infusion of high-dose meropenem (1–1.5 g q6h after 2 g LD) or imipenem (500 mg q4-6h after 1 g LD) are recommended as targeted therapy for the management of IVACs caused by carbapenem-susceptible *Acinetobacter baumannii* (Figure 2, panel B.1). Several clinical and in vitro evidence [69,70,71,72,73,74,75] demonstrated the efficacy of carbapenems (particularly meropenem and imipenem) for the treatment of carbapenem-susceptible *Acinetobacter baumannii* infections (Table 5).

Two different observational studies [69,70] compared imipenem with intravenous colistin for the management of critically ill patients affected by VAP due to MDR *Acinetobacter baumannii*, reporting no significant difference in clinical cure rate, mortality rate, resistance development, and adverse events between the two agents. Wang [71] retrospectively evaluated 30 critically ill patients affected by HAP/VAP due to MDR carbapenem-susceptible *Acinetobacter baumannii*, reporting a clinical cure rate of 100.0%. Furthermore, the administration of meropenem in EI was a cost-effective approach in this setting, although showing an equal clinical efficacy compared to intermittent infusion. Several in vitro studies [72,73,74,75] reported a variable susceptibility to carbapenems of *Acinetobacter baumannii* strains in different ecological settings, ranging from 41% to 75.8% and from 32.2% to 77.8% for meropenem and imipenem, respectively.

In regard to recommended dosages, evidence supporting the use of high-doses CI meropenem after 2 g LD stemmed from a PK/PD analysis carried out among 74 ICU patients affected by documented Gram-negative infections (of which pneumonia accounted for half of cases), in which a Css/MIC ratio ≥4.63 was significantly associated with favourable clinical outcome [68]. Monte Carlo simulation showed that, according to cumulative fraction of response, higher meropenem dosages by CI should be recommended for the management of *Acinetobacter baumannii* related infections among patients with preserved renal function [68]. Likewise, a similar aggressive PK/PD target of Css/MIC > 5 within the first 72 h was significantly associated with both microbiological eradication and prevention of resistance development in a large cohort of critically ill patients having documented Gram-negative infections treated with CI traditional beta-lactams [22].

#### 2.2.2. Carbapenem-Resistant *Acinetobacter baumannii*

Prolonged infusion of cefiderocol (2 g q8h EI or CI after 2 g LD) represents the first-line therapy in the management of IVACs caused by MDR *Acinetobacter baumannii*. Combination therapy including fosfomycin (6–8 g LD followed by 16–24 g/day CI), high-dose ampicillin-sulbactam (6 g/3g q8h CI after 6–8 g/3–4 g LD), and inhaled colistin (2 MU q8h) could be considered as second-line therapeutic alternative (Figure 2, panel B.2). A summary of the evidence assessing the efficacy of these antibiotic regimens in patients affected by IVACs caused by MDR *Acinetobacter baumannii* is provided in Table 6.

Although in vitro studies [44,45,63,79] reported high susceptibility rate of carbapenem non-susceptible *Acinetobacter baumannii* isolates against cefiderocol (MIC_50_ ranging from 0.12 mg/L to 0.25 mg/L, and susceptibility above the 85% in all different OXA-producing strains), clinical evidence are currently limited. In the CREDIBLE-CR RCT [41], a significant higher mortality rate in IVACs caused by *Acinetobacter baumannii* (accounting for 65% of HAP/VAP) was reported in patients treated with cefiderocol compared to best available therapy (49% vs. 18%; *p* = 0.04). In a case series including 13 patients, Gatti et al. [59] found a microbiological failure rate of 54% in critically ill patients affected by XDR *Acinetobacter baumannii* infections (84.6% VAP). Notably, microbiological failure occurred in 80% of patients with suboptimal *f*C_min_/MIC < 1 compared to 29% of those achieving optimal (*f*C_min_/MIC > 4) or quasi-optimal (*f*C_min_/MIC = 1–4) PK/PD target. In a case series, Falcone et al. [77] described two critically ill COVID-19 patients developing VAP caused by XDR *Acinetobacter baumannii* and treated with cefiderocol. Clinical failure was reported in 50% of cases. Conversely, Trecarichi et al. [78] reported a successful case of bacteraemic VAP caused by XDR *Acinetobacter baumannii* treated with cefiderocol as targeted therapy.

Different RCTs and observational studies [80,81,83] demonstrated the efficacy of high-dose sulbactam (up to 12 g/day) in monotherapy or combination therapy in IVACs caused by MDR *Acinetobacter baumannii*, showing no difference in clinical cure rate compared to colistin-based regimens [81]. However, evidence supporting combination therapy with fosfomycin are currently scanty. A case report [82] demonstrated the efficacy of combination therapy between high-dose sulbactam (9 g/day) and fosfomycin (16 g/day) in a critical care patients affected by meningitis due to MDR *Acinetobacter baumannii*. Notably, a synergism between sulbactam and fosfomycin was reported in 74% of MDR *Acinetobacter baumannii* isolates, resulting in a median MIC_50_ and MIC_90_ decrease up to eight-fold [83].

A systematic review including patients affected by HAP or VAP due to MDR *Acinetobacter baumannii* reported respectively a clinical success and microbiological eradication in 70.4% and 71.3% of cases with inhaled colistin monotherapy [65]. A retrospective case-control study [84] assessing 78 patients affected by respiratory infection or colonization due to MDR *Acinetobacter baumannii* found that the use of inhaled colistin was the only independent factor associated with microbiological eradication within 14 days after the index day (OR 266.33; 95% CI 11.26–6302.18, *p* < 0.001), and shortened the duration of MDR *Acinetobacter baumannii* recovery from the respiratory tract by 13.3 days.

In regard to recommended dosages, no evidence for administering cefiderocol by CI still exists Currently. However, it was shown in a descriptive case series of PK/PD target attainment and microbiological outcome in critically ill patients with documented severe XDR *Acinetobacter baumannii* BSI and/or VAP that treatment with cefiderocol with the standard 3 h infusion was ineffective in achieving the aggressive PK/PD of 100%fT > 4 × MIC in more than 50% of included patients. This resulted in a remarkable high rate of microbiological failure, especially among VAP cases [59].

Consequently, we recommend CI of cefiderocol after loading for rapidly achieving and then maintaining aggressive PK/PD targets in IVACs caused by *Acinetobacter baumannii*, also taking into account the limited ELF penetration rate of these agents [60,61,62].

## 3. Overview of Recommendations

Non-fermenting Gram-negative pathogens represent a leading cause of HAP or VAP in critically ill patients requiring mechanical ventilation [2]. Particularly, *Pseudomonas aeruginosa* represents one of the leading causative pathogens in critically ill patients affected by HAP or VAP [85]. The widespread diffusion of MDR and XDR isolates coupled with the emergence of high-risk clones (ST111, ST175, and ST235) calls for the prompt administration of adequate antibiotic therapy and optimization of lung exposure [23,85].

Two main cornerstones should be pursued in the management of IVACs caused by *Pseudomonas aeruginosa*: (1) the implementation of carbapenem-sparing regimens both in case of multi-susceptible isolates (considering the higher risk of relapse or resistance development [16,18]), preferring the use of piperacillin–tazobactam or third/fourth-generation cephalosporins (ceftazidime or cefepime), and of MDR/XDR isolates, favouring the administration of novel BL/BLIs (ceftolozane–tazobactam or ceftazidime–avibactam) or cefiderocol, according to the resistance mechanism exhibited by the specific strain); (2) the adoption of altered dosing strategies (high-doses and/or prolonged infusion) in order to achieve optimal PK/PD target at the site of infection, considering the limited pulmonary penetration of piperacillin–tazobactam, ceftazidime, cefepime, ceftazidime–avibactam, and cefiderocol (below 20%) [60,61,62,86,87,88]. Notably, in a prospective observational study including 72 patients affected by MDR *Pseudomonas aeruginosa* infections (79% ICU admission; 66.7% HAP/VAP), Plimis et al. [46] reported that intermittent infusion of ceftolozane–tazobactam was inadequate to achieve optimal PK/PD target for MIC ≥ 4 mg/L compared to continuous infusion. Similarly, ATS/IDSA guidelines recommended the use of prolonged infusion of piperacillin–tazobactam, ceftazidime, or cefepime in HAP or VAP due to *Pseudomonas aeruginosa* to maximize lung exposure [15].

The treatment of IVACs caused by MBL-producing *Pseudomonas aeruginosa* is challenging. In this scenario, cefiderocol could play a major role, although evidence is currently limited to only in vitro studies. The administration of inhaled colistin could represent a valuable therapeutic strategy in association with cefiderocol or meropenem and fosfomycin, providing a targeted antibiotic delivery in respiratory tract infections and resulting in lower systemic toxicity compared to intravenous colistin [89,90]. It should not be overlooked that after administration of a single 2 MU of inhaled colistimethate, colistin concentrations in the ELF ranged between 9.53 and 1137 mg/L, which are values much higher than that achievable after the administration of the same dose by the intravenous route (1.48–28.9 mg/L) [91]. The best dosing regimen of inhaled colistimethate has still to be defined, as quite variable dosages have been proposed in the literature, ranging from 1 MU q12h up to 5 MU q8h [91]. However, we believe that the dosage of 2 MU q8h should be preferred Currently considering that is the one supported by the major clinical evidence [65,91].

MDR or XDR *Acinetobacter baumannii* is a leading causative pathogen in critically ill patients affected by VAP, characterized by mortality rate up to 60% [92]. Although colistin- or polymyxin-based regimens were widely used in clinical practice for the management of severe *Acinetobacter baumannii* infections, relevant toxicity (mainly nephrotoxicity) and PK/PD issues (low ELF exposure, high occurrence of breakthrough infections) strongly affect their efficacy [92].

Notably, CREDIBLE-CR trial found [41] a remarkable mortality rate amongst patients affected by *Acinetobacter baumannii* infections, thus mitigating the initial expectations for cefiderocol [93]. Recently, it has been suggested to limit the use of cefiderocol to situations when intolerance or resistance to other generally active drugs has been shown [93]. However, the favourable safety profile, the high in vitro activity, and the potential maximization of lung exposure through the implementation of CI, make cefiderocol the first-line choice for targeted therapy of IVACs due to MDR *Acinetobacter baumannii* in critically ill patients.

By virtue of their lung penetration [94], fosfomycin could be a valid alternative for combination therapy with high-dose sulbactam (9–12 g/day) as second-line strategy for the management of MDR *Acinetobacter baumannii*. A recent observational study [95] identified fosfomycin-containing regimen as an independent predictor for 30-day survival in severe pneumonia caused by MDR *Acinetobacter baumannii*. However, none of these combination therapies included sulbactam.

Overall, in the management of IVACs caused by non-fermenting Gram-negative pathogens, the implementation of beta-lactams altered dosing strategies consisting in high-dose CI administration is strongly recommended for attaining aggressive PK/PD target of 100% fT > 4–8 × MIC and maximizing lung exposure [87,96,97]. This approach may both maximize clinical efficacy and prevent the development of resistance [87,98]. This aggressive strategy should be pursued also in the treatment of infections caused by wild-type strains. Optimal exposure into the ELF is difficult to be achieved and maintained with intermittent infusion of piperacillin–tazobactam, ceftazidime, and/or cefepime due to the limited penetration rates of these hydrophilic agents, and conversely, *Pseudomonas aeruginosa* was found to be an independent predictor of microbiological failure in critically ill patients affected by documented Gram-negative infections [22]. Clinicians must be aware that the antibiotic dosing regimens that we recommended throughout the manuscript are focused only on treatment of patients with normal renal function. It should not be overlooked that the pharmacokinetics of hydrophilic antimicrobial agents, namely beta-lactams and fosfomycin, may be affected among critically ill patients by several pathophysiological conditions that may alter volume of distribution and/or renal clearance [97,99]. Consequently, dose adjustments are needed in critically ill renal patients, especially among those with transient acute kidney injury, augmented renal clearance (ARC), and/or undergoing renal replacement therapy (RRT) [97,100]. In this scenario, adaptative daily therapeutic drug monitoring (TDM) may represent a valuable tool in addressing these issues. Currently, routinely TDM of BLs is strongly recommended for optimizing treatment among critically ill patients with Gram-negative infections [101], and an aggressive PK/PD target is considered useful among patients undergoing CRRT or having ARC [97]. Interestingly, the achievement of aggressive PK/PD target of Css/MIC ratio around 5 within the first 72 h of treatment with CI traditional beta-lactams was recently shown to be associated with significantly higher probability of both clinical outcome and microbiological eradication among critically ill patients with documented Gram-negative infections [22,68]. This suggests that early optimization of drug exposure by means of real-time TDM may be extremely helpful in maximizing treatment with beta lactams among the critically ill patients. However, it should be recognized that the extensive use of a real-time TDM-guided clinical pharmacologist advice approach is still burdened by many barriers [97,102,103,104]. To mention some of these, measurement of unbound concentrations, daily TDM assessment, timely turnaround time, appropriate interpretation of TDM data performed by well-trained MD or PharmD clinical pharmacologists, and implementation of user-friendly methods for novel beta-lactams still represent critical issues [97,102,103,104].

Furthermore, alternative agents should be considered for the treatment of patients with well-documented life-threatening beta-lactam allergies. Fluoroquinolones, aminoglycosides and colistin could be helpful in these cases depending on the susceptibility pattern of *Pseudomonas aeruginosa* or *Acinetobacter baumannii*. Besides, in case of life-threatening infections caused by MDR non-fermentative Gram-negatives with limited therapeutic options, desensitization protocols should be taken into consideration.

Finally, it should be mentioned that the current COVID-19 pandemic has led to a worldwide rise in antimicrobial resistance due to the massive disruption of infection control and antimicrobial stewardship measures in COVID ICUs. This caused a remarkable proportion of MDR bacterial co-infections and super-infections (including *Pseudomonas aeruginosa* and *Acinetobacter baumannii*) in severe COVID-19 patients [105]. In this scenario of more and more growing antimicrobial resistance, educational programs for improving the culture of knowledge of appropriate antibiotic use and the pivotal role of antimicrobial stewardship should be planned and delivered to young physicians [106].

In recent years, several leading guidelines for the management of Gram-negative infections have been issued by the most important Infectious Disease Societies worldwide [107,108,109]. Unfortunately, none of these focused on the treatment of IVACs and/or provided extensive recommendations for appropriate place in therapy of novel agents and/or for dosing optimization according to the PK/PD principles. Consequently, the implementation of developed therapeutic algorithms based on susceptibility pattern of non-fermenting Gram-negative isolated pathogens, coupled with the administration of altered dosing strategies of beta-lactams [97], could provide intensive care physicians a useful guidance for maximizing antibiotic treatment in ICU patients affected by IVACs, in order to address three main purposes: (a) to provide a personalized and targeted antibiotic therapy in each critically ill patient affected by HAP/VAP due to *Pseudomonas aeruginosa* or *Acinetobacter baumannii*; (b) to appropriately place novel antimicrobial agents in lack of definitive evidence; (c) to consider antimicrobial stewardship strategies for sparing the broadest-spectrum antibiotics (namely carbapenems). It is supposed that this strategy could maximize clinical outcome while minimizing resistance development in a challenging scenario as the management of IVACs in ICU patients.

## 4. Materials and Methods

A multidisciplinary task force, composed by one intensive care physician (B.V.), one infectious disease consultant (P.V.), one clinical microbiologist (G.M.R.), and one MD clinical pharmacologist (F.P.) met virtually on several occasions with the intent of developing algorithms for targeted antimicrobial therapy of IVACs caused by *Pseudomonas aeruginosa* and *Acinetobacter baumannii* in ICU critically ill patients. IVACs were defined as the presence of a ventilator-associated condition (consisting in a 48 h stable or decreasing daily minimum positive end-expiratory pressure [PEEP] or FiO_2_ followed by a rise in PEEP of 3 cm H_2_O or a rise in FiO_2_ of 0.2 sustained for 48 h) coupled with the occurrence of body temperature <36 °C or >38 °C and the start of at least one antibiotic agent continued for over 96 h. VAP was considered a subgroup of IVACs, consisting in the presence of at least 25 neutrophils/field coupled with positive semi-quantitative/quantitative culture for pathogenic organisms at bronchoalveolar lavage [110]. The definitive agreement for each therapeutic algorithm was reached by the multidisciplinary team after thoroughly discussion based on specific long-standing experience and on the specific expertise of each single member in terms of management of critically ill patients affected by IVACs, in appropriately placing in therapy of the old and novel antimicrobial agents, in implementing appropriate target antibiotic therapy and antimicrobial stewardship strategies in challenging scenarios, in applying traditional and novel microbiological methods and in interpreting microbiological findings and susceptibility test according to the specific clinical scenarios, and in optimizing and individualizing antibiotic dosing regimens according to the specific pathophysiological alterations. Each therapeutic algorithm was designed after that unanimous agreement among the four members of the multidisciplinary team was achieved. Six different scenarios were identified based on the resistance genotype of the pathogens and/or on the antibiotic susceptibility pattern (namely multi-susceptible *Pseudomonas aeruginosa*, MDR metallo-beta-lactamase-negative *Pseudomonas aeruginosa*, MDR metallo-beta-lactamase-positive *Pseudomonas aeruginosa*, carbapenem-susceptible *Acinetobacter baumannii*, and carbapenem-resistant *Acinetobacter baumannii*). MDR *Pseudomonas aeruginosa* isolates were defined according to the classification proposed by Magiorakos et al. [111]. A hierarchical scale was established whenever agreement on multiple therapeutic options was achieved in one specific scenario. Optimized antibiotic dosing schedules were provided as well.

A researcher (M.G.) retrieved the scientific evidence needed for supporting the specific choices included in the algorithms by means of a PubMed-MEDLINE literature search (until October 2021). Key terms were selected antibiotics, HAP, VAP, IVACs, and bacterial pathogens with genotype of resistance and/or antibiotic susceptibility pattern. Quality of evidence was established according to a hierarchical scale of the study design, as reported in the evidence pyramid [112]: randomized controlled trials (RCTs), prospective observational studies, retrospective observational studies, case series, case reports, and in vitro studies. International guidelines issued by the Infectious Disease Society of America and/or by the European Society of Clinical Microbiology and Infectious Diseases, systematic reviews and meta-analyses were also consulted. Consistence between retrieved studies was also considered, by assessing the concordance in clinical outcome of the included studies at each level of the evidence pyramid. Only articles published in English were considered, with a focus mainly on studies published in the last ten years.

## 5. Conclusions

In an era characterized by widespread diffusion of MDR Gram-negative pathogens and continuous increase in antibiotic resistance, the implementation of a multidisciplinary taskforce focusing on targeted therapy in critically ill patients has become a real need. Our approach is focused on prompt revision of inappropriate/unnecessary antibiotic therapy, implementation of “carbapenem-sparing” strategies, and PK/PD optimization of antibiotic exposure hopefully guided by real-time TDM whenever feasible. Rational use of broad-spectrum antibiotics, especially carbapenems, could represent a powerful strategy for tackling resistance spread in the ICU setting [14]. We believe that this strategy and these algorithms could be helpful in improving clinical outcomes and in avoiding resistance spread in the IVAC setting. The availability of molecular diagnostic tests that can rapidly provide information about the nature of the infecting pathogens and the presence of some relevant resistance determinants will be instrumental to improve antimicrobial stewardship practices based on the proposed algorithms.

## Figures and Tables

**Table 1:** Summary of the studies investigating the treatment of multi-susceptible *Pseudomonas aeruginosa* infection-related ventilator-associated complications (IVACs) with piperacillin–tazobactam or fourth-generation cephalosporins.

Author, Year and Reference	Study Design	No. of Patients	Antibiotic and Dosing	Rate of IVACs	Isolates	Severity	Clinical Outcomes	Relapse Rate— Resistance Development	Comments
**Piperacillin–tazobactam**									
Kalil et al., 2016 [15]	Guidelines	Piperacillin–tazobactam at dosage of 4.5 g q6h (preferring EI or CI) for empiric or definitive treatment of HAP/VAP caused by *Pseudomonas aeruginosa* according to antimicrobial susceptibility test (Strong recommendation; low-quality evidence)							
Jaccard et al., 1998 [16]	RCT, multicentre	371 (IMI vs. PIT)	IMI 500 mg q6h vs. PIT 4.5 g q8h	49.2% HAP	28% *P. aeruginosa*	Mechanical ventilation 47% APACHE II score: 14.6 ± 6.8	Clinical failure rate: 17% (PIT) vs. 29% (IMI) *p* = 0.09 Mortality rate for infection: 8% (PIT) vs. 9% (IMI) *p* = 0.78 Clinical failure rate in *P. aeruginosa* HAP: 10% (PIT) vs. 50% (IMI) *p* = 0.004	Resistance development 25.0% IMI vs. 4.8% PIT	PIT monotherapy is at least as effective and safe as IMI monotherapy in the treatment of HAP. In *P. aeruginosa* HAP, PIT achieved a better clinical efficacy than IMI, due to reduced development of microbiological resistance.
Joshi et al., 1999 [17]	RCT, multicentre	300 (155 PIT + tobramycin vs. 145 CTZ + tobramycin)	PIT 3.375 g q4h + tobramycin 5 mg/kg/day vs. CTZ 2 g q8h + tobramycin 5 mg/kg/day	87% HAP	7.7% *P. aeruginosa*	Severe infection 21%	Clinical cure rate: 74.2% (PIT) vs. 57.9% (CTZ) *p* = 0.004 Clinical cure rate in *P. aeruginosa* HAP: 67% (PIT) vs. 30% (CTZ) *p* = NS	NA	PIT plus tobramycin was shown to be more effective and as safe as CTZ plus tobramycin in the treatment of patients with HAP. A trend to higher microbiological eradication was found *P. aeruginosa* subgroup with PIT.
Babich et al., 2020 [18]	Retrospective, multicentre, propensity score adjusted analysis	767 (213 CTZ vs. 210 MER/IMI vs. 344 PIT)	All monotherapy 83.3% Intermittent infusion	All BSI 14.7% HAP/VAP	100% *P. aeruginosa* 7.6% MDR	ICU admission 16.6% Mechanical ventilation 12.1% SOFA score 4 (2–6)	Mortality rate: 17.4% (CTZ) vs. 20% (MER- IMI) vs. 16% (PIT) *p* = NS	Resistance development: 17.5% (MER-IMI) vs. 12.4% (CTZ) vs. 8.4% (PIT) *p* = 0.007	No significant difference in mortality, clinical, and microbiological outcomes or adverse events was demonstrated between CTZ, carbapenems, and PIT as definitive treatment of *P. aeruginosa* bacteraemia. Higher rates of resistance development were found in patients treated with carbapenems.
**Third/fourth-generation cephalosporins (Ceftazidime–Cefepime)**									
Kalil et al., 2016 [15]	Guidelines	Both ceftazidime and cefepime at dosage of 2 g q8h (preferring EI or CI) for empiric or definitive treatment of HAP/VAP caused by *Pseudomonas aeruginosa* according to antimicrobial susceptibility test (strong recommendation; low-quality evidence)							
Babich et al., 2020 [18]	Retrospective, multicentre, propensity score adjusted analysis	767 (213 CTZ vs. 210 MER/IMI vs. 344 PIT)	All monotherapy 83.3% Intermittent infusion	All BSI 14.7% HAP/VAP	100% *P. aeruginosa* 7.6% MDR	ICU admission 16.6% Mechanical ventilation 12.1% SOFA score 4 (2–6)	Mortality rate: 17.4% (CTZ) vs. 20% (MER- IMI) vs. 16% (PIT) *p* = NS	Resistance development: 17.5% (MER- IMI) vs. 12.4% (CTZ) vs. 8.4% (PIP-TZB) *p* = 0.007	No difference in mortality rate between ceftazidime and carbapenems at propensity score analysis (OR 1.14; CI 0.52–2.46). Significant higher occurrence of new resistance development in *P. aeruginosa* isolates in patients treated with carbapenems compared to ceftazidime (17.5% vs. 12.4%; *p* = 0.007).
Su et al., 2017 [19]	Retrospective	90	Cefepime 2 g q8h II	All BSIs 30% HAP/VAP	All *P. aeruginosa* cefepime-susceptible	ICU admission 32.2% Mechanical ventilation 25.6% Severe sepsis/septic shock 23.3% Mean APACHE II score: 22.07 Neutropenia 20%	Overall 30-day mortality rate: 36.7% Overall 30-day mortality rate in HAP/VAP subgroup: 59.3%	NA	A cefepime MIC of 4 mg/L may predict an unfavourable outcome among patients with serious infections caused by *P. aeruginosa*.
Ratliff et al., 2017 [20]	Retrospective, propensity score matched analysis	58 (29 MIC ≤ 2 mg/L vs. 29 MIC > 2 mg/L)	Ceftazidime 2 g q8h or Cefepime 2 g q12h	All BSIs 22.4% HAP/VAP	All *P. aeruginosa*	NA	30-day mortality rate: 17.2% vs. 27.6% (*p* = 0.34)	NA	No subgroup analysis was performed according to site of infection.

BSI: bloodstream infections; CTZ: ceftazidime; CI: continuous infusion; EI: extended infusion; HAP: hospital-acquired pneumonia; ICU: intensive care unit; IMI: imipenem–cilastatin; MER: meropenem; MIC: minimum inhibitory concentration NA: not available; OR: odds ratio; PIT: piperacillin–tazobactam; RCT: randomized controlled trial; VAP: ventilator-associated pneumonia.

**Table 2:** Summary of the studies investigating the treatment of multidrug-resistant (MDR) metallo-beta-lactamase-negative GES-negative *Pseudomonas aeruginosa* infection-related ventilator-associated complications (IVACs) with ceftolozane–tazobactam or cefiderocol.

Author, Year and Reference	Study Design	No. of Patients	Antibiotic and Dosing	Rate of IVACs	Isolates	Severity	Clinical Outcomes	Relapse Rate— Resistance Development	Comments
**Ceftolozane–tazobactam**									
Kollef et al., 2019 [24]	phase III RCT, multicentre (ASPECT-NP)	726 (362 CTT vs. 364 MER)	CTT 3 g q8h vs. MER 1 g q8h	All nosocomial pneumonia 71% VAP 6% secondary BSI	17.4% *P. aeruginosa* 38.1% MDR-PA 15.9% XDR-PA	ICU admission 92% APACHE II score ≥20 33% Median SOFA score 6 Median CPIS 10 Median duration of mechanical ventilation: 5 days	28-day mortality rate in *P. aeruginosa* subgroup: 25.4% vs. 18.5% (*p* = NS) Clinical cure rate 57.1% vs. 60% (*p* = NS)	NA	High-dose CTT is an efficacious and well tolerated treatment for Gram-negative HAP/VAP No difference in mortality and clinical cure rate for *P. aeruginosa* between CTT and MER, including MDR (54.2% vs. 54.5%) and XDR isolates (40% vs. 40%)
Pogue et al., 2019 [25]	Retrospective observational comparative, multicentre	200 (100 LOZ-TAZ vs. 100 polymyxin- or aminoglycoside-combination therapy)	CTT 1.5–3 g q8h vs. COL/polymyxin B or gentamycin— amikacin—tobramycin	52% VAP 12% HAP	100% *P. aeruginosa*	ICU admission 69% Mechanical ventilation 63% Severe sepsis/septic shock 42% Immunosuppression 14%	Clinical cure rate: 81% vs. 61% (*p* = 0.002) Overall AKI rate: 6% vs. 34% (*p* < 0.001) In-hospital mortality rate: 20% vs. 25% (*p* = 0.40)	Relapse 14% vs. 16% (*p* = NS)	CTT was independently associated with clinical cure (OR 2.63; 95% CI 1.31–5.30) and protective against AKI (OR 0.08; 95% CI 0.03–0.22) Preferential use CTT over polymyxins or aminoglycosides for MDR-PA infections.
Bassetti et al., 2019 [26]	Retrospective observational, multicentre (CEFTABUSE)	101	CTT 1.5–3 g q8h (CI/EI 18.8%) 38.6% first-line therapy	31.7% HAP/VAP	100% *P. aeruginosa* 17.8% MDR-PA 50.5% XDR-PA 2% PDR-PA	ICU admission 23.8% Mechanical ventilation 18.8% Septic shock 11.9% Solid organ transplant recipients 10.9% Haematological malignancy 12.9% Neutropenia 10.9%	Clinical cure rate: 83.2%	Relapse 7% Resistance 3%	Lower clinical success in patients with sepsis or requiring CRRT. Higher clinical failure (25.0) in pneumonia subgroup compared to other types of infection
Balandin et al., 2020 [27]	Retrospective observational, multicentre	95	CTT 1.5–3 g q8h	56.2% HAP/VAP 8.4% VAT	100% *P. aeruginosa* 48.4% XDR-PA 36.8% MDR-PA	ICU admission 100% Mechanical ventilation 80% Septic shock 45.3% RRT 27.4% Mean SOFA 6.9 Solid organ transplant recipients 6.2%	Microbiological eradication: 42.1% ICU mortality: 36.5%	Relapse 22.9%	Mortality rate in pneumonia subgroup: 34%
Fernandez-Cruz et al., 2019 [28]	Retrospective case-control	57 (19 CTT vs. 38 other agents)	CTT 3 g q8h (HAP/VAP or BSI) 84.6% targeted therapy	26.3% HAP/VAP	100% *P. aeruginosa* 52.6% MDR-PA 47.4% XDR-PA 100% ST-175 clone	ICU admission 26.3% Haematological malignancy 100% Neutropenia 63.2% Sepsis 15.8% Mean SOFA 5.42	14-day clinical cure rate: 89.5% vs. 71.1% (*p* = 0.18) 30-day mortality rate: 5.4% vs. 28.9% (*p* = 0.045)	Relapse 15.8%	CTT showed lower mortality compared to traditional therapy in severe PA infections in haematological patients. No subgroup analysis in patients with HAP/VAP was performed.
Gallagher et al., 2018 [29]	Retrospective observational, multicentre	205	CTT 1.5–3 g q8h Dose adjustment according to renal function	59% HAP/VAP	100% *P. aeruginosa*	ICU admission 51.2% Median APACHE II score 19 Solid organ transplant recipients 17.1%	Overall mortality rate: 19% Clinical cure rate: 73.7%	NA	Mortality rate was higher in VAP subgroup (37.9% vs. 19%) Clinical success was lower in VAP subgroup (50% vs. 73.7%) Pneumonia was associated with significant lower microbiological cure (OR 0.12; 95% CI 0.05–0.30)
Rodriguez-Nunez et al., 2019 [30]	Retrospective observational, multicentre	90	CTT 60% 3 g q8h	70% HAP/VAP 30% VAT	76.7% XDR-PA 23.3% MDR-PA Median MIC 2 mg/L	Septic shock 34.4% RRT 12.2% Solid organ transplant recipients 8.9%	30-day mortality rate: 27.8%	NA	MIC > 2 mg/L was an independent predictor of mortality at multivariate analysis
Munita et al., 2017 [31]	Retrospective observational	35	CTT 1.5–3 g q8h Dose adjustment according to renal function	51% HAP/VAP	100% CR-PA	NA	Overall clinical cure rate: 74%	NA	38.9% clinical failure rate in HAP/VAP subgroup
Haidar et al., 2017 [32]	Retrospective observational	21	CTT 1.5–3 g q8h	85.7% HAP/VAP	100% MDR-PA	Mechanical ventilation 38% Immunosuppression 43%	30-day mortality rate: 10% Clinical failure rate: 29%	Relapse 29% Resistance 14%	33.3% clinical failure rate in HAP/VAP subgroup
Bosaeed et al., 2020 [33]	Retrospective observational	19	CTT 1.5–3 g q8h	16% HAP 16% VAP	100% CR-PA	ICU admission 63% Haematological malignancy 26%	30-day mortality rate: 21% Microbiological eradication: 74%	NA	Microbiological failure in 50% of HAP/VAP cases
Diaz -Canestro et al., 2018 [34]	Prospective observational	58	CTT 1.5–3 g q8h 91.4% targeted therapy	60.3% HAP/VAP	86.2% XDR-PA 10.3% MDR-PA 50% ST-175 clone	ICU admission 27.6% Mechanical ventilation 32.8% Immunosuppression 12.1% Median SOFA 3	Clinical cure rate: 63.8% 30-day mortality rate: 27.6%	Resistance 13.8%	Clinical failure was documented in 42.9% of HAP/VAP ST-175 clone associated with higher risk of clinical failure at multivariate analysis
Escola-Verge et al., 2018 [35]	Retrospective observational	38	CTT 1.5–3 g q8h	36.8% HAP/VAP	100% XDR-PA Median CTT MIC: 2 mg/L	ICU admission 31.6% Solid organ transplant recipients 28.9% Neutropenia 15.8%	90-day clinical cure: 68.4% 90-day mortality rate: 13.2%	Relapse 21.1%	Clinical failure in HAP/VAP subgroup: 25%
Xipell et al., 2018 [36]	Retrospective observational	23	CTT	17.4% HAP 17.4% VAT	79% XDR-PA 17% MDR-PA 4% PDR-PA	NA	Clinical cure rate: 87.5% 6-weeks mortality rate: 21.7%	NA	Higher mortality rate in respiratory tract infections (37%). Significant higher mortality rate in patients with HAP/VAP treated with low-dosage (1.5 g q8h) vs. high-dose (3 g q8h) CTT (60% vs. 0%)
Caston et al., 2017 [37]	Case series	12	CTT 100% targeted therapy	50% HAP/VAP	100% MDR-PA	Septic shock 83.3%	Overall mortality rate: 25% Microbiological eradication: 83.3%	Resistance: 16.6%	Mortality rate in HAP/VAP subgroup: 33.3%
Dinh et al., 2017 [38]	Case series	15	CTT Median daily dose 6 g/day	46.7% nosocomial pneumonia (85.7% VAP)	100% XDR-PA	ICU admission 53.3% Mean SOFA score 7.6 Immunosuppression 66.7%	Clinical failure: 33.3% In-hospital mortality rate: 27%	Relapse 11.1%	Clinical failure in nosocomial pneumonia subgroup: 40%
Gelfand et al., 2015 [39]	Case series	3	CTT 3 g q8h	100% VAP	100% MDR-PA	ICU admission 100% Mechanical ventilation 100%	Clinical cure: 100%	NA	
Hakki et al., 2018 [40]	Case series	6	CTT 3 g q8h	50% pneumonia	100% MDR-PA	Haematopoietic-cell transplant recipients 100%	Clinical cure rate: 66.7%	Relapse 28.6%	33.3% clinical failure rate in patients with nosocomial pneumonia
**Cefiderocol**									
Bassetti et al., 2020 [41]	Phase 3, randomized, prospective, multicentre, open-label (CREDIBLE-CR)	150 (101 cefiderocol vs. 49 BAT)	Cefiderocol 2 g q8h (3h-infusion) 100% target therapy Dose adjustment according to renal function	44.6% HAP/VAP	15% *P. aeruginosa*	ICU admission 56% Septic shock 19% Mechanical ventilation 50% Immunocompromised 27% Mean SOFA score 5.1	Mortality rate in PA subgroup: 35% vs. 17% (*p* = NS) Clinical cure at the end of treatment (HAP/VAP subgroup): 60% vs. 63%	NA	A numerically higher proportion of patients with CRE infections achieved a clinical cure in the cefiderocol group than in the BAT group (66% vs. 45%).
Wunderink et al., 2020 [42]	Phase 3, randomized, prospective, multicentre, open-label (APEKS-NP)	300 (148 cefiderocol vs. 152 meropenem)	Cefiderocol 2 g q8h (3 h infusion) vs. MER 2 g q8h (3 h infusion)	123 VAP 119 HAP 50 HCAP	16.4% *P. aeruginosa* 8% carbapenemase-producers	ICU admission: 68% Mechanical ventilation: 60% Mean SOFA score 4.8 APACHE II score ≥ 16: 49%	Mortality rate at 14-day in PA subgroup: 8% vs. 13% (*p* = NS) Clinical cure rate in PA subgroup: 67% vs. 71% (*p* = NS)	NA	Cefiderocol was non-inferior to high-dose, extended-infusion MER in terms of all-cause mortality on day 14 in patients with Gram-negative nosocomial pneumonia
Delgado-Valverde et al., 2020 [43]	In vitro study	6	5 ST-175; 1 IMP+. Cefiderocol MIC range: 0.125–0.5 (100% susceptibility)						
Mushtaq et al., 2020 [44]	In vitro study	111	30 VIM+; 25 IMP+; 20 GES+; 15 PER+; 11 NDM+; 10 VEB+. Overall resistance rate (MIC > 2): 18.9%. Susceptibility: VIM 93.3%; GES 90.0%; VEB 90.0%; IMP 80.0%; PER 66.7%; NDM 45.5%.						
Kazmierczak et al., 2019 [45]	In vitro study	353	321 carbapenemase-negative meropenem non-susceptible (MIC range 0.002–8; MIC_50_ 0.12; MIC90 1); 26 VIM+ (MIC range 0.008–2; MIC_50_ 0.25; MIC90 2); 4 IMP+ (MIC range 1–2); 4 GES+ (MIC range 0.12–0.25)						

AKI: acute kidney injury; BAT: best available therapy; BSI: bloodstream infections; CI: continuous infusion; COL: colistin; CR: carbapenem-resistant; CRE: carbapenem-resistant Enterobacteriaceae; CRRT: continuous renal replacement therapy; CTT: ceftolozane–tazobactam; EI: extended infusion; HAP: hospital-acquired pneumonia; HCAP: healthcare-associated pneumonia; ICU: intensive care unit; MER: meropenem; MIC: minimum inhibitory concentration; MDR: multidrug-resistant; NA: not available; NS: not significant; OR: odds ratio; PA: *Pseudomonas aeruginosa*; PDR: pan drug-resistant; RRT: renal replacement therapy; SOFA: sequential organ failure assessment; VAP: ventilator-associated pneumonia; VAT: ventilator-associated tracheitis; XDR: extensively drug-resistant.

**Table 3:** Summary of the studies investigating the treatment of multidrug-resistant (MDR) metallo-beta-lactamase-negative GES-positive *Pseudomonas aeruginosa* infection-related ventilator-associated complications (IVACs) with ceftazidime–avibactam monotherapy or in combination with fosfomycin.

Author, Year and Reference	Study Design	No. of Patients	Antibiotic and Dosing	Rate of IVACs	Isolates	Severity	Clinical Outcomes	Relapse Rate—Resistance Development	Comments
**Ceftazidime–avibactam**									
Torres et al., 2018 [48]	Phase III RCT, multicentre (REPROVE)	808 (405 CTV vs. 403 MER)	CTV 2.5 g q8h vs. MER 1 g q8h	67% HAP 33% VAP	30% *P. aeruginosa* CTV MIC_90_: 8 mg/L	Mechanical ventilation 43%	Overall clinical cure: 68.8% vs. 73% (*p* = NS) Clinical cure in PA subgroup: 64.3% vs. 77.1% (*p* = NS)	NA	CTV potential alternative to carbapenems in the management of nosocomial pneumonia, also caused by PA
Jorgensen et al., 2019 [49]	Retrospective observational, multicentre	63	CTV 2.5 g q8h Dose adjustment according to renal function	60.3% HAP/VAP	100% *P. aeruginosa* CTV MIC_50_: 2 mg/L CTV MIC_90_: 6 mg/L	ICU admission 55.6% Median SOFA score 5 Immunocompromised 6.3%	Clinical failure: 30.2% 30-day mortality rate: 17.5%	Relapse 6.3% Resistance 0%	CTV could be an effective therapy for MDR-PA as well as CRE infections. No difference in mortality rate between PA and CRE treated with CTV (17.5% vs. 16.2%; *p* = NS)
Vena et al., 2020 [50]	Retrospective observational, multicentre	41	CTV 2.5 g q8h (36.6% CI/EI) 80.5% targeted therapy Dose adjustment according to renal function	48.8% nosocomial pneumonia (65% VAP—35% HAP)	80.5% *P. aeruginosa*	ICU admission 41.5% Mechanical ventilation 34.1% Septic shock 17.1% CRRT 12.2% Solid organ transplant recipients 19.5% Haematological malignancies 9.8% Neutropenia 12.2%	Clinical success in HAP/VAP: 90% Clinical cure rate in PA subgroup: 87.8%	NA	CTV as value option for XDR-PA infection, including HAP/VAP
Rodriguez-Nunez, 2018 [51]	Case series	8	CTV 2.5 g q8h Dose adjustment according to renal function	62.5% HAP/VAP	MDR/XDR PA	NA	Clinical cure rate: 50% (40% in HAP/VAP subgroup) 30-day mortality rate: 37.5% (60% in HAP/VAP subgroup)	Relapse 20%	
Santevecchi et al., 2018 [52]	Case series	3	CTV 2.5 q8h Dose adjustment according to renal function	100% VAP	2 MDR-PA 1 XDR-PA	ICU admission 100% Mechanical ventilation 100%	Clinical cure rate: 66.7%	None	
Xipell et al., 2017 [53]	Case report	1	CTV 2.5 g q8h	HAP	XDR-PA	NA	Clinical cure 100%	None	
Recio et al., 2018 [54]	In vitro analysis of a retrospective study	24	CTV	33.3% HAP/VAP	All XDR-PA 45.8% GES-5-positive ST235 clone 41.1% VIM-2 ST175 clone 13.1% non-carbapenemase producers	Overall susceptibility rate to CTV in GES-5-positive strains:100%; MIC_90_ 6 mg/L CTV demonstrated in vitro high activity against GES-positive strains			
**Ceftazidime-Avibactam + Fosfomycin**									
Papp -Wallace et al., 2019 [55]	Preclinical study— murine model infection	The association between CTV and FOS significantly reduced the *P. aeruginosa* (CFUs), by approximately 2 and 5 logs, compared with stasis and in the vehicle-treated control, respectively. Administration of ceftazidime–avibactam and fosfomycin separately significantly increased CFUs, by approximately 3 logs and 1 log, respectively, compared with the number at stasis, and only reduced CFUs by approximately 1 log and 2 logs, respectively, compared with the number in the vehicle-treated control. The combination of CTV + FOS was superior to either drug alone and has the potential to offer infected patients with high bacterial burdens a valid therapeutic choice against infection with MDR-PA that lack metallo-beta-lactamases.							
Avery et al., 2019 [56]	In vitro study	53	CR-PA: CTV baseline susceptibility 89.5%. Synergism with FOS in 25% of isolates (FICI ≤ 0.5)						
Mikhail et al., 2019 [57]	In vitro study	21	MDR-PA. CTV MIC reduction in 13/21 (61.9%) of isolates in combination with FOS. Combination between CTV and FOS was indifferent at time-kill analysis.						

CFU: colony format unit; CI: continuous infusion; CR: carbapenem-resistant; CRE: carbapenem-resistant Enterobacteriaceae; CRRT: continuous renal replacement therapy; CTV: ceftazidime–avibactam; EI: extended infusion; FICI: fractional inhibitory concentration index; FOS: fosfomycin; HAP: hospital-acquired pneumonia; ICU: intensive care unit; MER: meropenem; MIC: minimum inhibitory concentration; MDR: multidrug-resistant; NA: not available; NS: not significant; PA: *Pseudomonas aeruginosa*; RCT; randomized controlled trial; SOFA: sequential organ failure assessment; VAP: ventilator-associated pneumonia; XDR: extensively drug-resistant.

**Table 4:** Summary of the studies investigating the treatment of multidrug-resistant (MDR) metallo-beta-lactamase-positive *Pseudomonas aeruginosa* infection-related ventilator-associated complications (IVACs) with cefiderocol in association with inhaled colistin or combination therapy between meropenem, fosfomycin and inhaled colistin.

Author, Year and Reference	Study Design	No. of Patients	Antibiotic and Dosing	Rate of IVACs	Isolates	Severity	Clinical Outcomes	Relapse Rate—Resistance Development	Comments
**Cefiderocol**									
Mushtaq et al., 2020 [44]	In vitro study	66	30 VIM+; 25 IMP+; 11 NDM+. Susceptibility: VIM 93.3%; IMP 80.0%; NDM 45.5%.						
Kazmierczak et al., 2019 [45]	In vitro study	30	26 VIM+ (MIC range 0.008–2; MIC_50_ 0.25; MIC_90_ 2); 4 IMP+ (MIC range 1–2).						
Jacobs et al., 2019 [63]	In vitro study	27	VIM+ (number of isolates not reported); MIC range 0.03–1; MIC_50_ 0.25; MIC_90_ 0.5						
**Meropenem + Fosfomycin + inhaled colistin**									
Albiero et al., 2019 [64]	In vitro study	19	10 MBL+. Synergism was found in 100% of isolates with a FICI ≤ 0.5. Median reduction in MIC50 and MIC90 by 8-fold. PK/PD simulation showed that 6–8 g q8h FOS achieved the probability of target attainment of ≥90% at an MIC of 32 mg/L. 1.5 g q6h MER in 3 h EI achieved the probability of target attainment of ≥90% at an MIC of 16 mg/L. Combination therapy significantly increase the cumulative fraction rate against MBL-PA compared to monotherapy with MER (32% vs. 68%) or FOS (0% vs. 74%).						
**Inhaled colistin**									
Vardakas et al., 2018 [65]	Systematic review and meta-analysis	12 studies including 373 patients (8 VAP–2 HAP–2 VAT)	MDR-PA and MDR-AB mainly investigated. Pooled all-cause mortality: 33.8%; clinical success 70.4%; eradication of Gram-negative pathogens 71.3% of cases.						

AB: *Acinetobacter baumannii*; EI: extended infusion; FICI: fractional inhibitory concentration index; FOS: fosfomycin; HAP: hospital-acquired pneumonia; MBL: metallo-beta-lactamase; MER: meropenem; MIC: minimum inhibitory concentration; MDR: multidrug-resistant; PA: *Pseudomonas aeruginosa*; PK/PD: pharmacokinetic/pharmacodynamic; VAP: ventilator-associated pneumonia; VAT: ventilator-associated tracheitis.

**Table 5:** Summary of the studies investigating the treatment of carbapenem-susceptible *Acinetobacter baumannii* infection-related ventilator-associated complications (IVACs) with carbapenems.

Author, Year and Reference	Study Design	No. of Patients	Antibiotic and Dosing	Rate of IVACs	Isolates	Severity	Clinical Outcomes	Relapse Rate—Resistance Development	Comments
**Carbapenems (Meropenem–Imipenem)**									
Garnacho -Montero et al., 2003 [69]	Prospective observational	35 (21 colistin vs. 14 imipenem)	Colistin 2.5–5 mg/kg/day in three doses vs. Imipenem 2–3 g/day in three/four doses	100% VAP	21 carbapenem -resistant AB 14 carbapenem- susceptible AB	ICU admission 100% Septic shock 57.1% APACHE II score: 20.5 ± 7 SOFA score: 11.7 ± 6.6	Clinical cure rate: 57% vs. 57% (*p* = NS) Mortality rate: 61.9% vs. 64.2% (*p* = NS) VAP-related mortality rate: 38.0% vs. 35.7% (*p*=NS)	NA	No difference in efficacy and safety between carbapenem and intravenous colistin in the management of VAP caused by MDR-AB
Kallel et al., 2007 [70]	Retrospective matched case-control	120 (60 colistin vs. 60 imipenem)	Colistin 2 MU q8h vs. Imipenem 500 mg q6h	100% VAP	61.7% carbapenem- susceptible AB 38.3% carbapenem- susceptible *P. aeruginosa* (in patients receiving imipenem)	ICU admission 100% SAPS-II 33.2 ± 10.8 Septic shock 23.3%	Clinical cure rate: 75% vs. 71.7% (*p* = 0.68) Mortality rate: 41.7% vs. 35% (*p* = 0.45)	Relapse: 8.3% Resistance development: 0.0%	No difference in efficacy and safety between carbapenem and intravenous colistin in the management of VAP caused by MDR-AB
Wang, 2009 [71]	Retrospective, observational monocentric	30	MER 1 g q8h 1 h infusion vs. MER 500 mg q6h 3 h infusion	100% HAP	100% MDR cabapenem- susceptible AB	ICU admission 100% Mechanical ventilation 100%	Clinical cure rate at day 7: 100.0% vs. 100.0% (*p* = NS)	Relapse rate: 3.3% Resistance development: 0.0%	EI treatment with MER is a cost-effective approach for the management of HAP due to MDR-AB, being equally clinically effective to II
Ikonomidis et al., 2006 [72]	In vitro study	320	40.6% resistance to meropenem (MIC_50_ 4 mg/L; MIC_90_ 8 mg/L); 67.8% resistance to imipenem (MIC_50_ 8 mg/L MIC_90_ 64 mg/L)						
Mezzatesta et al., 2008 [73]	In vitro study	107	88.8% MDR-AB. 59% resistance to meropenem (MIC_90_ 64 mg/L); 50% resistance to imipenem (MIC_90_ 32 mg/L)						
Guzek et al., 2013 [74]	In vitro study	54	22.2% resistance to doripenem; 22.2% resistance to imipenem; 42.6% resistance to meropenem						
Jones et al., 2005 [75]	In vitro study	33	100% wild-type Acinetobacter spp isolates. 75.8% susceptibility to meropenem (MIC_90_ > 8 mg/L); 75.8% susceptibility to imipenem (MIC_90_ > 8 mg/L); 75.8% susceptibility to doripenem (MIC_90_ 16 mg/L)						

AB: *Acinetobacter baumannii*; EI: extended infusion; HAP: hospital-acquired pneumonia; ICU: intensive care unit; II: intermittent infusion; MER: meropenem; MDR: multi-drug resistant; MIC: minimum inhibitory concentration; NA: not available; NS: not significant; RCT: randomized controlled trial; VAP: ventilator-associated pneumonia.

**Table 6:** Summary of the studies investigating the treatment of carbapenem-resistant *Acinetobacter baumannii* infection-related ventilator-associated complications (IVACs) with cefiderocol or combination therapy between fosfomycin and ampicillin/sulbactam.

Author, Year and Reference	Study Design	No. of Patients	Antibiotic and Dosing	Rate of IVACs	Isolates	Severity	Clinical Outcomes	Relapse Rate—Resistance Development	Comments
**Cefiderocol**									
Bassetti et al., 2020 [41]	Phase 3, randomized, prospective, multicentre, open-label (CREDIBLE-CR)	150 (101 cefiderocol vs. 49 BAT)	Cefiderocol 2 g q8h (3h-infusion) 100% target therapy Dose adjustment according to renal function	44.6% HAP/VAP	65% *A. baumannii*	ICU admission 56% Septic shock 19% Mechanical ventilation 50% Immunocompromised 27% Mean SOFA score 5.1	Mortality rate in AB subgroup: 49% vs. 18% (*p* = 0.04) Clinical cure at the end of treatment (HAP/VAP subgroup) :60% vs. 63%	NA	A significant higher mortality rate in patients affected by AB infections was found with cefiderocol compared to BAT
Gatti et al., 2021 [59]	Case series	13	Cefiderocol 1.5–2 g q8h	84.6%	100% XDR-AB	100% ICU admission 100% mechanical ventilation	30-day mortality rate: 30.8% Microbiological failure: 54%	NA	Microbiological failure occurred in 80% of patients with suboptimal *f*C_min_/MIC compared to 29% of those achieving optimal or quasi-optimal *f*C_min_/MIC ratio.
Bavaro et al., 2021 [76]	Case series	13	Cefiderocol 2 g q8h (3h-infusion) Dose adjustment according to renal function	7.7%	76.9% Carbapenem-resistant-AB 15.4% XDR-PA 7.7% KPC	38.5% ICU admission	30-day mortality rate: 23.1% Microbiological eradication: 100.0%	NA	Combination therapy with fosfomycin was successfully implemented in 9 cases, including VAP due to carbapenem-resistant AB
Falcone et al., 2020 [77]	Case series	10	Cefiderocol 1.5–2 g q6-8h	2 VAP	2 XDR *Acinetobacter baumannii*	ICU admission 100% Mean SOFA score 10 CRRT 50%	Clinical failure rate in AB VAP: 50% 30-day mortality rate in AB VAP: 50%	No relapse in AB VAP	Cefiderocol suggests that it may be useful to treat unresponsive ICU-acquired infections due to MDR AB
Trecarichi et al., 2019 [78]	Case report	1	Cefiderocol 2 g q8h (3h-infusion) target therapy	VAP/BSI	XDR–*Acinetobacter baumannii*	ICU admission Mechanical ventilation Septic shock	Clinical cure	NA	
Hackel et al., 2017 [79]	In vitro study	173 MER-non susceptible (US) 595 MER-non susceptible (EU)	MIC range 0.002–8; MIC_50_ 0.25; MIC_90_ 1 MIC range 0.004-64; MIC_50_ 0.12; MIC_90_ 1						
Mushtaq et al., 2020 [44]	In vitro study	99	41 OXA-23; 20 NDM; 19 OXA-51; 10 OXA-58; 9 OXA-24/40. Susceptibility: 94.7% OXA-51; 90% OXA-58; 88.9% OXA-24/40; 85.4% OXA-23; 50% NDM						
Kazmierczak et al., 2019 [45]	In vitro study	768	543 OXA-23; 124 OXA-24; 86 carbapenemase-negative/MER non-susceptible; 14 OXA-58; 7 GES; 2 NDM. MIC range 0.002-64; MIC_50_ 0.12; MIC_90_ 1						
Jacobs et al., 2019 [63]	In vitro study	101	Carbapenem non-susceptible isolates: MIC range 0.03–>64; MIC_50_ 0.25; MIC_90_ 1 (96.0% susceptibility)						
**Fosfomycin + Ampicillin/Sulbactam**									
Betrosian et al., 2007 [80]	RCT	27	AMS 6 g/3 g q8h vs. AMS 8 g/4 g q8h	100% VAP	MDR *Acinetobacter baumannii*	ICU admission 100% Mechanical ventilation 100% Mean APACHE II score 15	Clinical cure rate: 64.3% vs. 69.2% (*p* = 0.79) 30-day mortality rate: 42.9% vs. 53.8% (*p* = NS)	NA	The use of high-dose AMS regimens is effective for the treatment of VAP caused by MDR-AB.
Betrosian et al., 2008 [81]	Prospective observational	28 (15 COL vs. 13 AMS)	AMS 6 g/3 g q8h vs. COL 3 MU q8h	100% VAP	MDR *Acinetobacter baumannii*	ICU admission 100% Mechanical ventilation 100% Mean APACHE II score 14	Clinical cure rate: 61.5% vs. 60% (*p* = NS) 28-day mortality rate: 30% vs. 33% (*p* = NS)	NA	COL and high-dose AMS were comparably safe and effective treatments for critically ill patients with MDR *A. baumannii* VAP.
Mellon et al., 2012 [82]	Case report	1	AMS 3 g/1.5 g q4h + FOS 4 g q6h	Meningitis	MDR *Acinetobacter baumannii* MIC AMS 32 mg/L	ICU admission	Clinical cure	NA	The only case reporting the clinical efficacy of combination therapy between fosfomycin and high-dose sulbactam for the management of deep-seated AB infection.
Mohd Sazlly Lim et al., 2021 [83]	In vitro study	50	Fosfomycin in combination with sulbactam showed synergism in 74% of AB isolates, resulting in a median MIC_50_ and MIC_90_ reduction respectively of 4–8-fold.						
**Inhaled colistin**									
Vardakas et al., 2018 [65]	Systematic review and meta-analysis	12 studies including 373 patients (8 VAP—2 HAP—2 VAT)	MDR-PA and MDR-AB mainly investigated. Pooled all-cause mortality: 33.8%; clinical success 70.4%; eradication of Gram-negative pathogens 71.3% of cases.						
Kuo et al., 2012 [84]	Retrospective, case-control	78 (39 inhaled colistin + other antibiotics with activity against AB vs. 39 other antibiotics with activity against AB)	Inhaled COL	41% HAP/VAP 59% respiratory colonization	100% MDR-AB	ICU admission 71.8% Mechanical ventilation 38.5% RRT 7.7% APACHE II score 20.0 ± 6.2	Microbiological eradication at 14-day: 84.6% vs. 10.3% (*p* < 0.001) 28-day mortality rate: 12.8% vs. 10.3% (*p* = 0.72)	Relapse rate: 21.2% COL MIC increase: 28.6%	The use of inhaled COL was the only independent factor associated with the eradication of MDR-AB within 14 days after the index day (OR 266.33; 95% CI 11.26–6302.18, *p* < 0.001), and shortened the duration of MDR-AB recovery from the respiratory tract by 13.3 ± 1.45 days.

AB: *Acinetobacter baumannii*; AMS: ampicillin-sulbactam; BAT: best available therapy; BSI: bloodstream infections; COL: colistin; CRRT: continuous renal replacement therapy; FOS: fosfomycin; HAP: hospital-acquired pneumonia; ICU: intensive care unit; MIC: minimum inhibitory concentration; MDR: multidrug-resistant; NA: not available; NS: not significant; RCT: randomized controlled trial; RRT: renal replacement therapy; SOFA: sequential organ failure assessment; VAP: ventilator-associated pneumonia; VAT: ventilator-associated tracheitis; XDR: extensively drug-resistant.

## Data Availability

The data presented in this review are retrieved and summarized from the different publicly available included studies.
